# EEG Characteristics to Hyperventilation by Age and Sex in Patients With Various Neurological Disorders

**DOI:** 10.3389/fneur.2021.727297

**Published:** 2021-09-22

**Authors:** Irma Khachidze, Manana Gugushvili, Maia Advadze

**Affiliations:** ^1^Department of Human Psychophysiology, I. Beritashvili Centre of Experimental Biomedicine. Tbilisi, Georgia; ^2^Faculty of Medicine, Georgian National University SEU, Tbilisi, Georgia

**Keywords:** EEG, HPT, patients with CNS disorder, pathological EEG responses to hyperventilation, age and sex dependency

## Abstract

**Introduction:** Hyperventilation provocation test(s) (HPT) concomitant to electroencephalography (EEG) may detect hidden disorders of the nervous system (CNS). There are various types of abnormal EEG in responses to HPT that provoke different interpretations. However, it is not evident how the onset time of pathological EEG responses to hyperventilation (PERH) reveals dysfunction of the CNS in humans. It is also not clear if age and biological sex affect EEG characteristics in response to HPT. Our previous studies have revealed three types of PERH (disorganization of basic rhythm, paroxysmal discharges, epileptiform activity) concerning the manifestation time of first, second, and third minutes. The current work aims to classify the PERH with regards to age (3–6, 7–12, 13–18, 19–30, 31–50, 50 > year) and the biological sex of the patients.

**Methods:** This study examined the EEG of 985 outpatients with various functional disorders of the CNS. The patients were assigned to one of three experimental groups based on the time occurrence of PERH in response to the HPT.

**Results:** The disorganized basic EEG rhythm in the first, second, third minute of HPT was observed across all age and sex groups. All three types of PERH in the first minute were comparable for both sexes. However, some discrepancies between females compared to males were observed in the second and third minutes. All three types of PERH in the first and the second minutes were found only in women. The second type of PERH has revealed at the second minute of PHT in 13–18-year-old five girls.

**Conclusion:** The three main types of PERH were detected at the first minute in all age groups and sex in patients with various CNS dysfunctions. It is diagnostically informative should be used as a marker during the monitoring of treatment. The specific activity of the brain's response to HPT depends on time, age, sex. The data indicate that taking into account sex differences and age during HPT leads to better results. The sensitivity and severity of the NS reaction toward hypocapnia, stress, and emotion increase in women. Therefore, in such cases should not be recommended to expand functional loads.

## Introduction

Hyperventilation provocation test(s) (HPT) is often used for the detection of hidden disorders of the nervous system and the pathological forms of the Central Nervous System (CNS) in humans (1). The HPT implies fast breathing for 3 (2, 3) or 3–5 min (4, 5). This method is used to diagnose hidden forms of epilepsy among other dysfunctions of the CNS such as migraine, neuropathy, epidemic encephalitis, etc. (6).

Further, it is accepted that electroencephalography (EEG) is an objective and reliable method for describing normal and pathological processes of the CNS (7, 8). HPT presented during EEG might advance the diagnostic value of epilepsy (9, 10) and increase the accuracy of the results in a clinical study (11–15) and scientific research for various neurological disorders (16–21). The practice proves that the EEG responses to hyperventilation are heterogeneous (1, 22, 23). Moreover, various types of abnormal EEG responses to HPT provoke different interpretations. However, it is not evident how pathological EEG responses to hyperventilation (PERH) may reveal dysfunction of the CNS in humans. Although it is well-established that brain processes during development contribute to the manifestation and/or efficacy of treatments of brain disorders, data on the response to HPT in people with various functional disorders of the CNS is limited (9, 24, 25). Additionally, only a few studies described the role of age and biological sex (female, male) (26, 27) in patients with CNS dysfunctions. Some studies of the HPT either didn't explore the age effect (14, 28) or didn't specify developmentally meaningful age groups (i.e., 3–6, 7–12, 13–18, 19–30, 31–50, 51 years and above) and provided evidence only for youth (7), adults (26, 27), or above 10 years (12, 29). It is also not clear if age and biological sex affect EEG characteristics in response to HPT. This type of research and interpretation of the results will be essential and informative in evaluating and investigating neurobiological mechanisms of neurodevelopmental and neurodegenerative disorders in men and women with different age ranges. In our previous studies, we described pathological EEG responses to hyperventilation in individuals with various CNS dysfunctions (23, 24, 30). Three types of pathological responses: I, II, and III were documented. We also detected the time-specific manifestation of abnormal response on the first, second, and third minutes of hyperventilation. The current study aims to fill the gap on age- and sex-specific EEG response on HPT in outpatients with various CNS dysfunctions.

## Materials and Methods

### Study Participants

The sample used for this study has been described elsewhere (23, 24, 30). Briefly, in our previous studies, 2,186 patients were recruited according to the EEG responses to hyperventilation. The control group consisted of 1,201 participants whose EEG response to hyperventilation was within the normal range. The three types of PERH (I, II, and III) were detected in 985 outpatients; PERH-I corresponds to disorganization of basic rhythm without paroxysm. PERH-II—high-amplitude, generalized, monomorphic or polymorphic, slow, synchronous waves with paroxysmal discharges without epileptic elements. PERH-III was shown epileptic activity, both with generalized paroxysmal discharges and individual graph elements of epilepsy (23, 24, 30). The diagnosis was made according to neurological examination and clinical assessment by medical doctors and collected from clinical history. These EEGs were recorded in the Beritashvili Centre of experimental Biomedicine (IBCEB) and Tatishvili Medical Centre.

The EEG of outpatients with pathological EEG to HPT was re-evaluated concerning age and sex. The current work was the classification of PERH, taking into account the age and biological sex of the patients.

### The Studied Groups

#### Temporal Profile of PERH Manifestation

The patients were assigned to one of three experimental groups based on the time occurrence of PERH in response to the HPT. Group A: the PERH in the first minute of HPT; Group B: the PERH in the second minute; Group C: the PERH in the third minute.

#### Age and Sex

The patients were grouped by developmentally meaningful age (3–6, 7–12, 13–18, 19–30, 31–50, 51 years, and above). Studied participants were stratified by biological sex as well (female, male).

#### EEG

The EEG tests were conducted following international performance standards. It was a part of the prescribed therapy plan after obtaining consent forms signed by the patients and their parents when appropriate (i.e., children and adolescence).

#### The EEG Recording and Methods of Analysis

The EEGs were always conducted in the morning. An EEG test without a functional load (3 min in length) preceded the EEG recording with a functional load. The functional trials were performed with a 3-min long PHT with open and closed eyes, and the breath-hold (15–25 s); the recording finished with closed eyes. The average EEG recording was 35–40 min.

The EEG signals were digitally recorded using a set of 19 scalp electrodes according to the International 10–20 system (Am. EEG Society 10-20 EEG Placement. https://www.ers-education.org) (31) and ENCEPHALAN 131-03, professional version “MEDICOM.” The bandpass of the amplifiers was 0.5–100 Hz, and the notch filter was 50 Hz. The signals from each input electrode were digitized with a sampling rate of 256 Hz with a resolution of 12 bits. Electrode (Ag/AgCl) specific resistance was not higher than 5 kΩ.

Artefact-free EEG epochs were selected−7 fragments for each patient.

#### The Quantitative Assessment of the EEG Epochs

A fast Fourier transformation algorithm of signal processing was used to obtain the power spectrum for each lead within six frequency bands: Delta (0.5–4.0 Hz), Theta-1 (4.0–6.0 Hz), Theta-2 (6.0–8.0 Hz), Alpha (8–13 Hz), Beta-1 (13–24 Hz) Beta-2/Gamma (24–50.8 Hz). Following features of the EEG response to hyperventilation were assessed: wave amplitude; correspondence of the indices and topography of given age group; area of predominance; type of low-frequency oscillations, their regularity, availability, or absence rhythmicity; the degree of synchronization and stability.

#### The Qualitative Assessment of the EEG Characteristics

The qualitative assessment carried out corresponding age standards (Interictal epileptiform abnormalities: the presence of epileptiform activity (spike discharges, sharp waves, paroxysmal burst); the number of paroxysmal discharges indicating abnormal EEG activity; characteristics of low frequency, alpha, beta activity, the regularity of the rhythm, frequency stability, gradients, domination area, the symmetry of the amplitude and frequency, the amplitude limits and stability (9, 32).

Ethical approval was obtained from the IBCEB Ethics Committee and the study adhered to the Declaration of Helsinki (1964).

#### Data Management and Statistical Analysis

After preliminary evaluation of selected epochs of EEG, the tables and figures for the changes in EEG characteristics (dynamics) from each of 19 standard recording electrodes were organized for each patient. Statistical significance for the difference in variables registered at of the investigation was assessed using Mann-Whitney *U*-test (BIOSTAT). The changes in the EEG characteristics for the whole group were assessed by the Wilcoxon Signed-Ranks test (33). It was reported in our previous studies (23, 24, 30). Data analysis was done using the software package of statistical data processing SPSS 20.0 to check and evaluate the result. The Chi-square test was used to compare variables and differences. The *p* < 0.05 was considered to be significant.

## Results

The study participants had range of neurodevelopmental, neurodegenerative, psychiatric disorders (epilepsy 51%, encephalopathy 21%, attention deficit, hyperactivity disorder-17%, Cerebral palsy-9%, autism spectrum disorders-3%, headaches 42%, fatigue 14%, anxiety 29%, sleep disorders 11%, stroke-14%, alzheimer-9%, schizophrenia-2%).There was an overlap of some symptoms between disorders. The distribution of patients by time manifestation of PERH and biological sex showed in tables and figures.

PERH was revealed in 853 participants in group A (the first minute of hyperventilation), that is 86.6% of the survey participants, in group B (second minute of hyperventilation)- in 95 (9.54%), and group C (third minute of hyperventilation)-in 37(3.8%) (Table 1).

**Table 1:** Distribution of patients by the time manifestation of PERH and biological sex.

**Gender**	**Number of patients**	**Group A**	**Group B**	**Group C**
Male	432 (43.9%)	371(85.9%)	43 (9.9%)	18 (4.2%)
Female	553 (56.1%)	482 (87.2%)	52 (9.4%)	19 (3.4%)
Total	985 (100%)	853 (86.6%)	95 (9.6%)	37 (3.8%)

The results showed types of PER based on the age and biological sex of the patients. Nine hundred eighty-five (985) patients divided by PERH types the following age groups: 3–6 year−130 (13.2%), 7–12 year−264 (26.8%), 13–18 year 167 (17%), 19–30 year 163 (16.5%), 31–50 years 175 (17.8%), 50-year and above 86 (8.7%) patients.

All three types of PERH-I, PERH-II, PERH-III, were revealed on EEG during the first minute of the HPT irrespective of age and sex. Further, PERH-I i.e., disorganization of the main rhythm was the predominant, compared to PERH- II and PERH- II was predominant compared to PERH-III (*p* < 0.0001).

### The Types of PERH as a Function of Manifestation Time in Response to HPT

#### Group A (First Minute of Hyperventilation)

**Figure 1:**
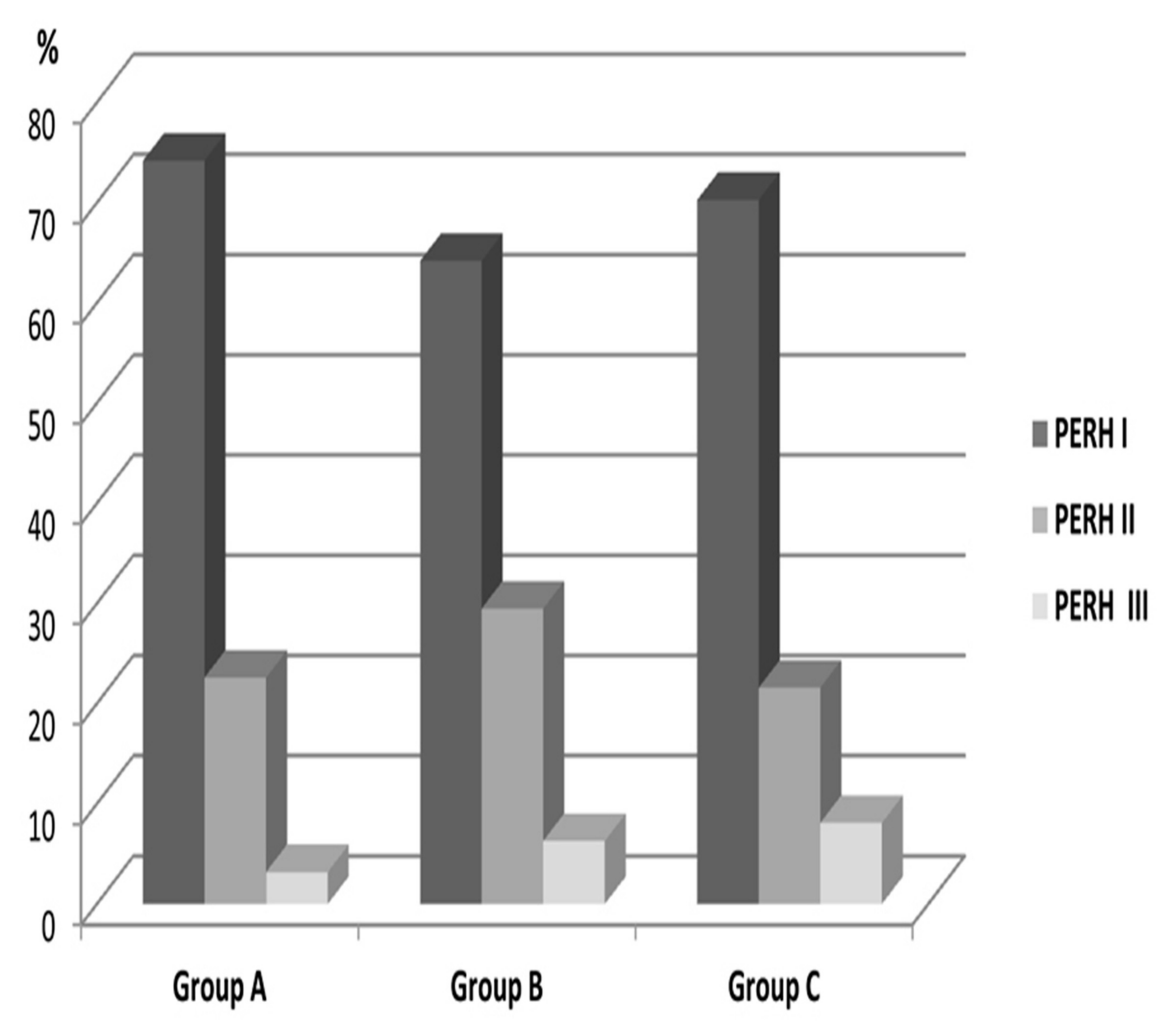
Distribution of patients by PERH types frequency and the time of manifestation in the group A, B, C. PERH-I (disorganization of basic rhythm) is the first column, PERH-II (paroxysmal discharges) is second column, PERH-III (epileptic activity) is the third column. PERH-I was observed in the first, second and third minutes of HPT in all patients irrespective of age and sex. PERH-I was the predominant, compared to PERH- II and PERH- II was predominant, compared to PERH-III.

There was a significant difference in PERH type frequency *p* < 0.0001; $\chi_{\left(2\right)}^{2}$ = 689. 8. PERH-I in 633 (74.2%) patients, RERH-II in 193 (22.6%), and PERH-III in 27 (3.2%) patients (Figure 1, Group A).

#### PERH by Age

**Figure 2:**
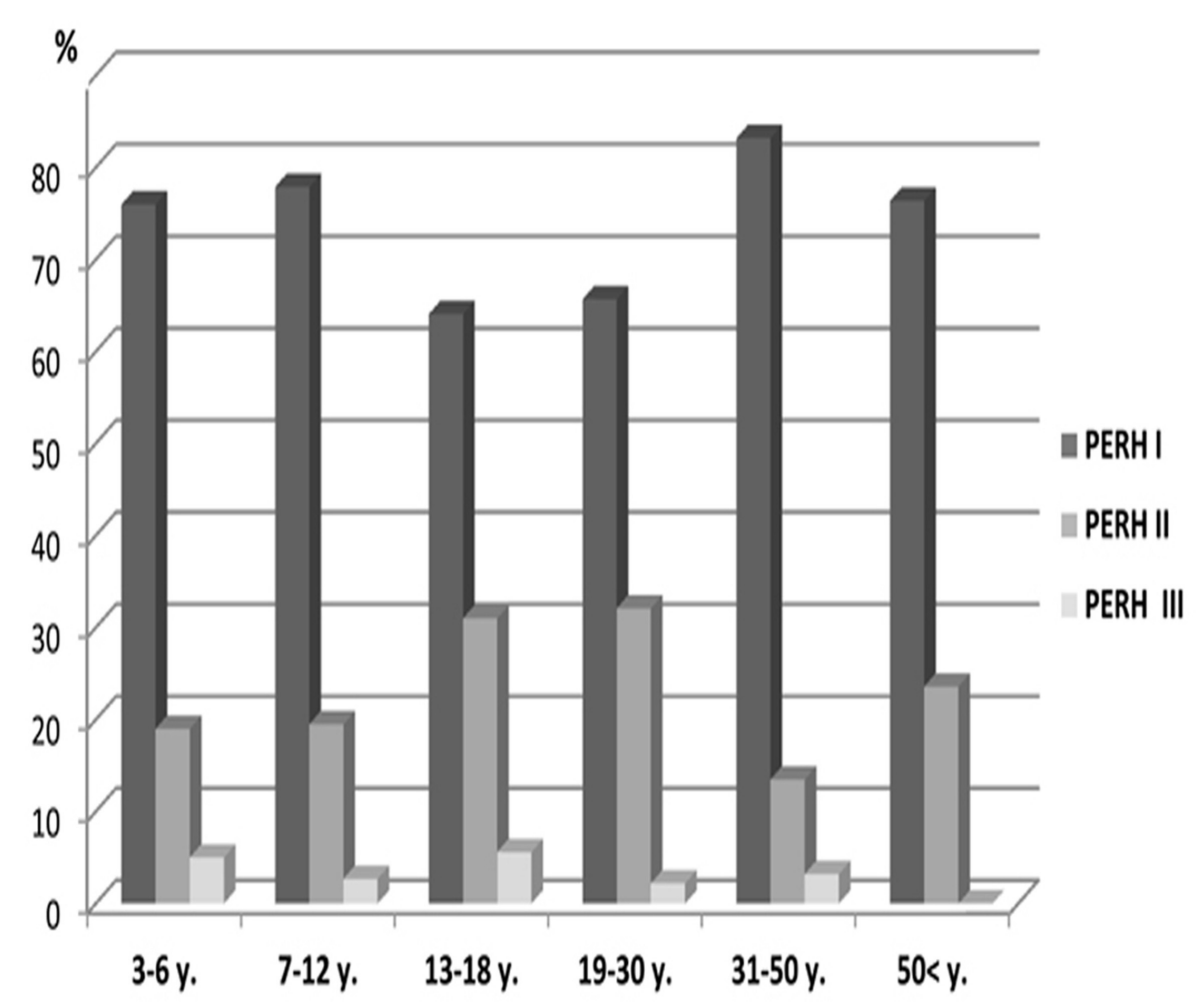
Frequency distribution (%) of patients by pathological EEG responses to hyperventilation (PERH) types for various age groups in Group A. A majority of patients developed PERH-I followed with PERH-II (*p* < 0.05) irrespective of age.

The data of patients with PERH - I, II, and III were significant by age *p* < 0.002; $\chi_{\left(10\right)}^{2}$ = 28.2 (Figure 2). PERH-I was predominant, compared to PERH- II and PERH- II was predominant compared to PERH-III. A majority of patients developed PERH-I followed with PERH-II (*p* < 0.05) irrespective of age.

Frequency distribution (%) of patients by pathological EEG responses to hyperventilation (PERH) types for various age groups reported in Table 2.

**Table 2:** Frequency distribution of patients by PERH type and age in Group A.

**EEG Type**	**3–6 year**	**7–12 year**	**13–18 year**	**19–30 year**	**31–50 year**	**50 year >**
PERH- I	92 (76%)	176 (77.9%)	91 (84.1%)	90 (65.7%)	129 (83.2%)	55 (76.4%)
PERH- II	23 (19%)	44 (19.5%)	44 (31.0%)	44 (32.2%)	21 (13.5%)	17 (23.6%)
PERH-III	6 (5%)	6 (2.6)	7 (4.9%)	3 (2.2%)	5 (3.2%)	0 (0%)
SUM	121 (100%)	226 (100%)	142 (100%)	137 (100%)	155 (100%)	72 (100%)

#### PERH by Sex

**Figure 3:**
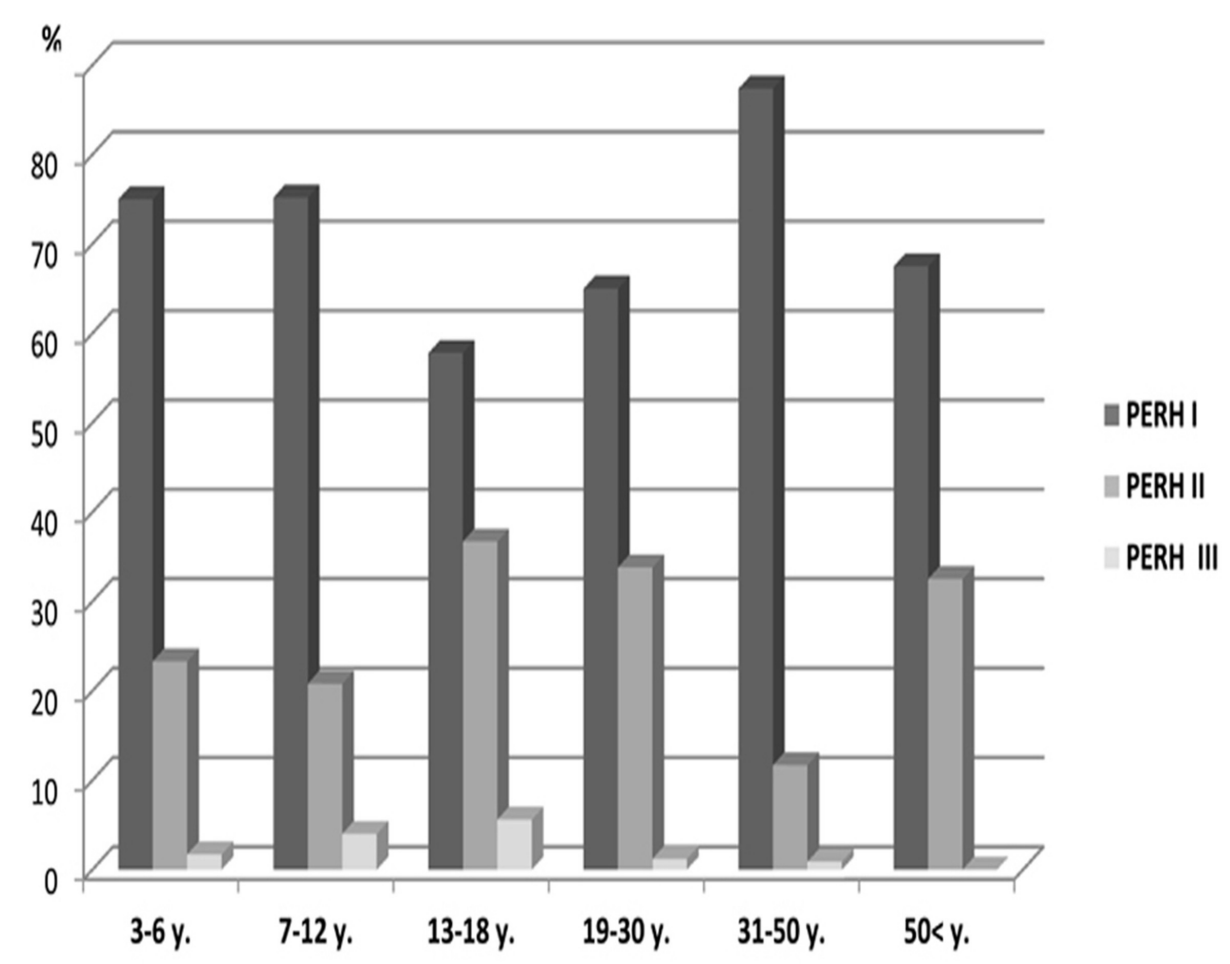
Frequency distribution (%) of female patients by pathological EEG responses to hyperventilation (PERH) types for various age groups in Group A. A majority of female patients developed PERH-I followed with PERH-II (*p* < 0.05) irrespective of age.

Study investigated 371 males and 482 females (Table 1, Group A). PERH-I was the predominant, compared to PERH- II and PERH- II was predominant compared to PERH-III in females. PERH was revealed in females *p* < 0.001; $\chi_{\left(10\right)}^{2}$ = 30.9. Study participants were grouped by PERH type and age in females. Figure 3 reported the frequency distribution of female patients by PERH types for various age groups. A majority of female patients developed PERH-I followed with PERH-II irrespective of age.

### Group B (Second Minute of Hyperventilation)

The PERH was found in 95 (96%) patients: PERH types frequency were significant *p* < 0.0001; $\chi_{\left(2\right)}^{2}$ = 48.4. PERH-I was predominant, compared to PERH- II and PERH- II was predominant compared to PERH-III. PERH-I prevailed in 61 (64, 2%) patients. PERH-II was in 28 (29 4%) patients, PERH-III-in 6 (6.3%) patients (Figure 1, Group B).

#### PERH by Age

**Figure 4:**
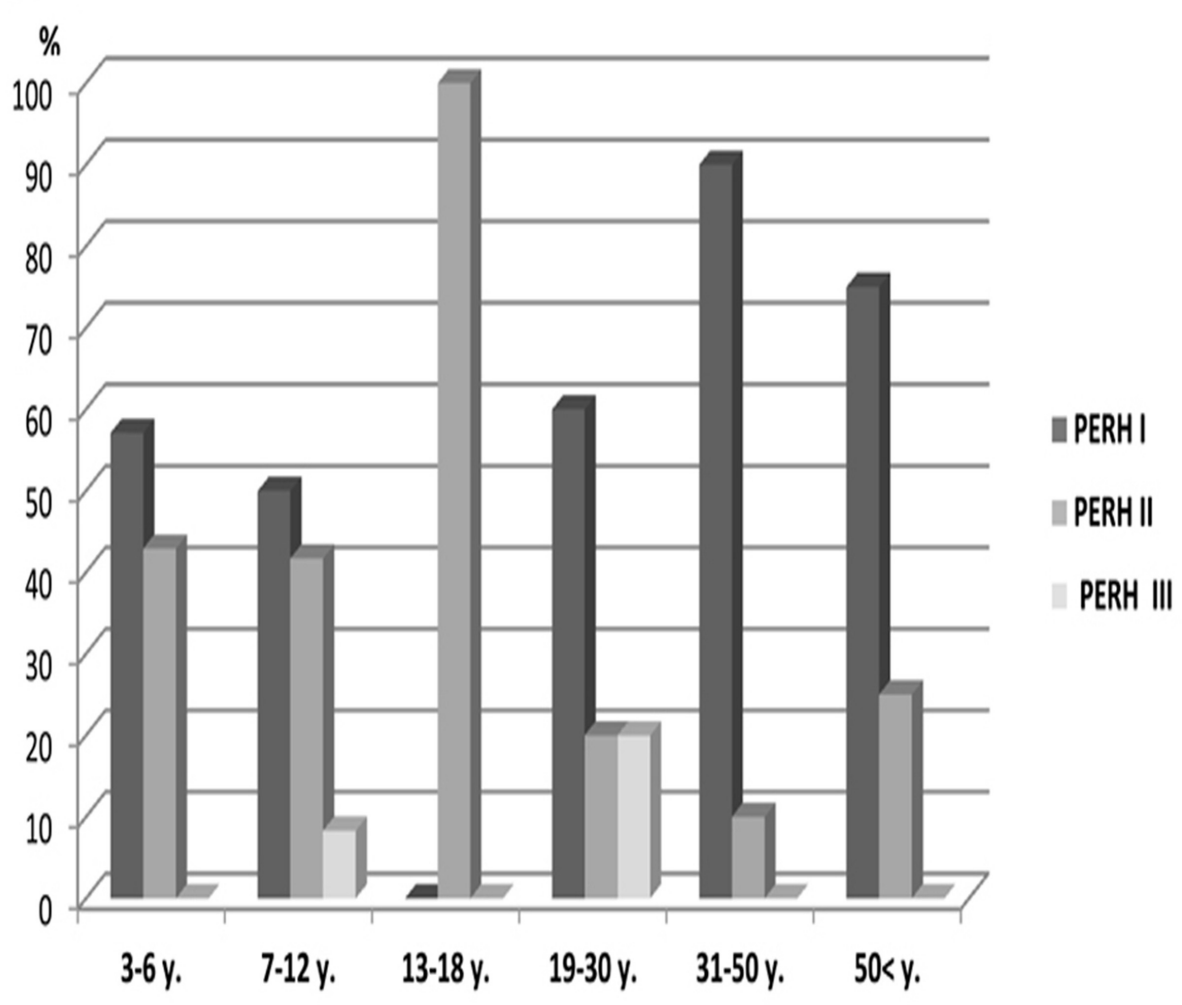
Frequency distribution (%) of female patients by pathological EEG responses to hyperventilation (PERH) types for various age groups in Group B. A majority of female patients developed PERH-I followed with PERH-II (*p* < 0.05) irrespective of age.

**Figure 5:**
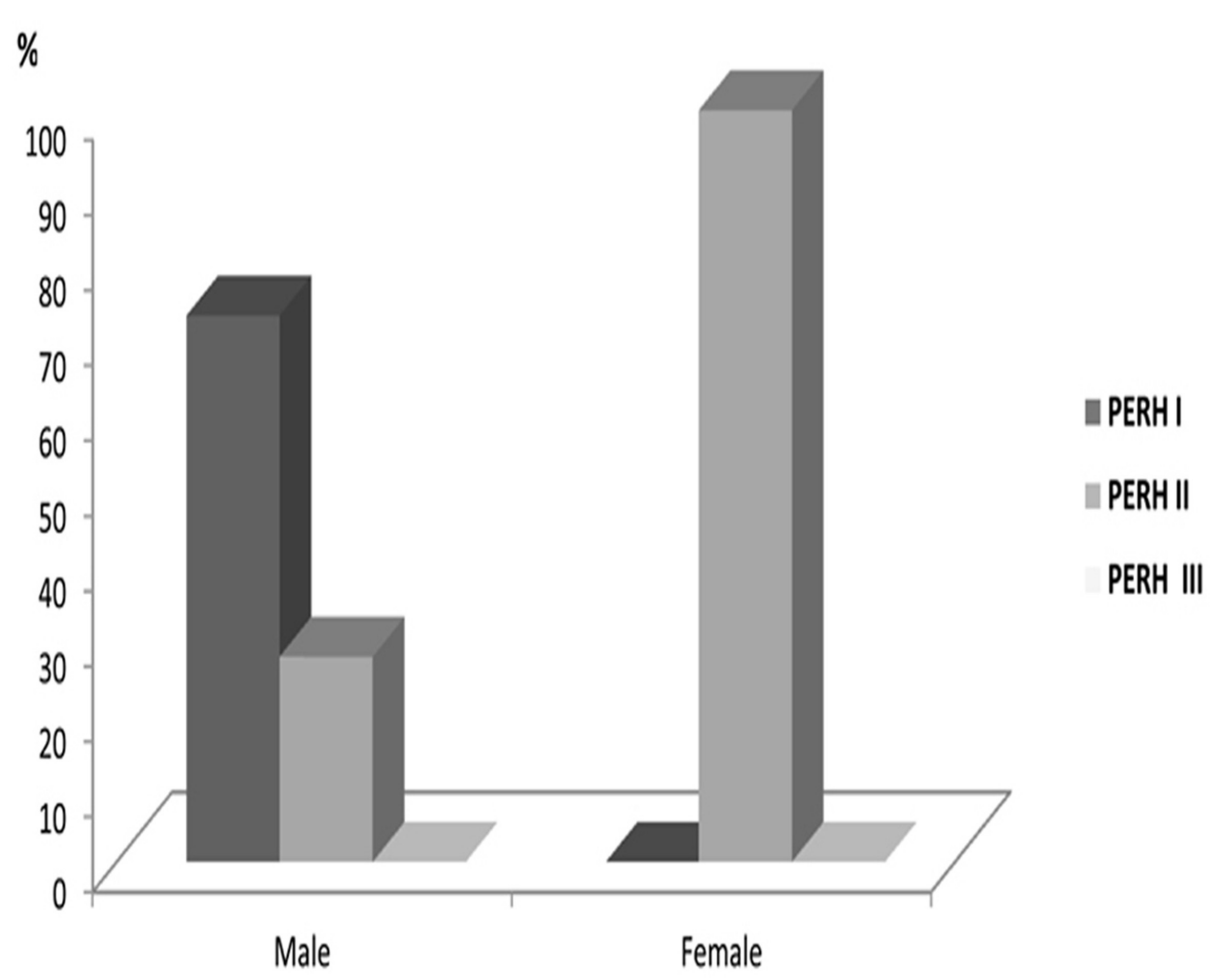
Comparison of 13–18-year-olds male and female patients by PERH types in Group B. The PERH- I is high in males, while in the females revealed PERH II.

There was no statistically significant PERH types difference across age groups (*p* > 0.05) ***PERH by sex*** The study investigated 43 males and 52 females in Group B (Table 1, Group B). In females, PERH was statistically significant *p* < 0.035; $\chi_{\left(10\right)}^{2}$ = 19.5. PERH was revealed in females compared to males *p* < 0.072; $\chi_{\left(10\right)}^{2}$ = 17.1. Figure 4 reported the frequency distribution of female patients by PERH types for various ages. A majority of female patients developed PERH-I followed with PERH-II irrespective of age. The PERH-I primarily dominated in all age groups of females except 13–18-year-olds, showed a different EEG pattern, they predominantly had PERH II (paroxysmal discharges) *p* < 0.019; $\chi_{\left(2\right)}^{2}$ = 7.9. Comparison of 13–18-year-old males and females by PERH showed in Group B. The PERH- I primarily dominated in males while the females revealed PERH II *p* < 0.007; $\chi_{\left(1\right)}^{2}$ = 7.3 (Figure 5).

### Group C (Third Minute of Hyperventilation)

The PERH was found in 37 patients. The PERH types were significant *P* < 0.0001; $\chi_{\left(2\right)}^{2}$ = 23.7. PERH-I was the predominant compared to PERH- II and PERH- II was predominant compared to PERH-III. PERH-I was detected in 26 (70.3%) patients, PERH-II-in 8 (21.6%), and PERH-III- in 3 (8.1%) patients. EEG predominantly revealed the disorganization of basic rhythmicity (Figure 1, Group C).

#### PERH by Age and Sex

The study investigated 18 males and 19 females (Table 1, Group A). The statistical analysis according to the PERH types, age, and sex was not significant (*p* > 0.05).

## Discussion

The study considers the detection of the pathological types of EEG response to hyperventilation (PERH) in patients with various CNS dysfunctions regarding three factors—the time of manifestation, age, and sex. In particular, the discretions of EEG responses to HPT in the first, second, and third minutes, by age (3–6, 7–12, 13–18, 19–30, 31–50, 51, and above) and biological sex (woman and men) of patients.

PERH predominantly revealed at the first minute of HPT that it is diagnostically informative and should have prognostic value. These findings are consistent with the data of other authors (1, 22), whose note that EEG changes are observed at the beginning of forced breathing at the first minutes of hyperventilation. Correlation between the PERH, age, and gender at the third minute of hyperventilation was not observed in our research. Accordingly, the prolongation of HPT for the next minutes is inadvisable in patients with various disorders, especially for children (18, 23, 34). We saw disorganization of basic EEG rhythm in the first, second and third minutes of hyperventilation. The reaction of CNS to hyperventilation test to induce different changes such as the intensity of breathing, the glucose level in the blood, a patient's posture (sitting position) has been reported (4, 35). Based on the EEG observations, a group of researchers (36–38) indicated that children (under 12 years) were particularly vulnerable to hypocapnia developed in response to hyperventilation. Our results showed that the sensitivity to hyperventilation was high in all ages and sex. In the first minute of HPT, were revealed three types of PERH in all ages. It was not observed in the second and third minutes. Based on this, we can assume that one of the factors predisposing the high sensitivity of the central nervous system to hyperventilation is the age of the individual. The EEG reaction to hyperventilation undergoes permanent changes during brain maturation and development. Different studies widely describe it (22, 39, 40). We should point out that children, adults, and the elderly have different sensitivity to the hypocapnia developed during hyperventilation (36, 41). In our previous (23, 30, 34, 37) and current studies, this has been revealed in patients with various CNS dysfunctions. The EEG response to hyperventilation, besides the time of manifestation and age, was studied regarding the biological sex of patients. Scientific data are scarce on the response to forced breathing in individuals of different age groups, both in women and men with CNS disorders. The characteristics of EEG activity related to sex are not described. Works that indicated the age and gender of patients in the EEG response to hyperventilation sex specificity and differences are not mentioned (8, 10, 29). We suppose that the study is informative to compare brain maturation and development in healthy and pathological conditions in women and men. In our work, the three types of PERH in the first and the second minutes were found in all age groups only in women. The PERH by sex was not observed in the third minute. Our data related to the effects of hyperventilation during the puberty period in subjects with various CNS dysfunctions, particularly in women, are considerable. Generalized high-amplitude monomorphic and polymorphic low-wave activity with synchronic paroxysmal discharges without epileptiform elements revealed at the second minute of hyperventilation only in 13–18-year-old women. The paroxysmal reaction of the brain (PERH-II) is more complex than the disorganization of its basic rhythm (PERH-I). Our study revealed gender-specific brain development and sensitivity to hyperventilation in 13–18-year-old women patients. The correlation of 13–18-year-old women and men from group B (second minutes) revealed a significant difference in the type of EEG reaction; in women detected paroxysmal activity, in contrast to men. We hypothesized that brain sensitivity toward hypocapnia increases during puberty, especially in girls and adolescents. Our result is based on a small number (5 women), and we will collect additional data for a comparative study and extend and strengthen observations. We are planning, that should address this issue in future studies when the number of patients we'll be increased. Nevertheless, the major conclusions of this study are not solely based on this subgroup of participants. Therefore, we don't state the findings for the second minute of HPT as the key finding of this study. Instead, we argue that PERH-I in response to the HPT was prevalent in the first and the second minutes of HPT only in women, irrespective of age.

Based on various MRI studies, the children with normal brain development show the difference in brain maturation related to gender and age (42). Girls reach white and gray matter maturation 1–2 years earlier than boys (43, 44). MRI results by other authors related to differences in age, gender, brain volume. Also, prove that the growth of white matter in women occurs at a higher rate than in men (45, 46). The investigations related to functional features of brain maturation using EEG shown differences between girls and boys. Gender-related EEG differences in 7–10-year-old children indicate the prevalence of brain maturity in girls compare to boys (40, 44). The study confirmed the increase in alpha rhythm dominant frequency and modulation in 7-year-old girls. We can say that brain maturation in children and adolescents with CNS disorders is delayed compared to age. It is generally observable in the female group (especially 13–18-year-old). The response to HPT in different physiological and pathological conditions might advance our understanding of neurobiological processes in various CNS diseases. We would like to increase the number of participants for some studied disorders. In addition to the role of time, age and sex, another possible impact of other cofounder factors could influence PERH (as individual sensitivity and type of CNS disorders). The possible impact of other cofounder factors (e.g., individual sensitivity and type of CNS disorders), in addition to the role of time of manifestation, age and sex, should be interesting. Regretfully, the richness of data does not allow discussing all observations in one manuscript.

The presented research is significant as the description/comparison of the type of EEG modifications response to HPT in patients with different neurological and psychiatric conditions. The study considers all three factors (time of detection, the patient's age, and sex). In its turn, it allows for a more detailed and objective analysis of the hyperventilation effect to NS that will have scientific value and clinical application. Understanding mechanisms underlying brain electrical activity is crucial for explaining many illnesses and conditions. Particularly the identification of brain electrogenesis on hyperventilation in cases of various CNS dysfunctions may have a wide range of practical and theoretical interest in different fields like neuroscience, neuropharmacology, neuropsychology, psychiatry, neurology (different types of cerebral-vascular disorders), cardiovascular pathology, COVID-19 affect, pulmonology, sports medicine, and other spheres.

## Conclusion

Three main types of PERH were detected at the first minute in all ages and sex of patients with various CNS dysfunctions. It is diagnostically informative should be used as a marker during the monitoring of treatment. We assume that the electrical activity of the brain response to HPT depends on the time, age, and sex of patients. We hope our data indicate that taking into account sex differences and age during HPT leads to better results. The sensitivity and severity of the NS reaction toward hypocapnia, stress, and emotion increase in women (especially during puberty). Therefore, in such cases should not be recommended to expand functional loads.

## Study Limitations and Recommendations

The study has some limitations. The study sample was relatively small in the second minute of HPT (B group) with only five girl representatives aged 13–18. It is recommended to study a larger sample to ensure reliability and reflect better this group population. We will be able to collect additional data in the future for a comparable study and extend and strengthen observations reported in this manuscript. Nevertheless, the major conclusions of this study are not solely based on this subgroup of participants.

## Data Availability Statement

The datasets used and analyzed during the current study will be made available by the corresponding author upon reasonable request.

## Ethics Statement

The studies involving human participants were reviewed and approved by I. Beritashvili Center of Experimental Biomedicine, Ethics Committee. Written informed consent to participate in this study was provided by the participants' legal guardian/next of kin.

## Author Contributions

IK: study design, analysis, and interpretation of obtained data. MG: statistical analysis and technical preparation of the manuscript. MA: data collection and ethical issue. All authors agree to be accountable for the content of the work.

## Funding

Article co-funded by Vetenskapsrådet Swedish Research Links, no. 2016-05871.

## Conflict of Interest

The authors declare that the research was conducted in the absence of any commercial or financial relationships that could be construed as a potential conflict of interest.

## Publisher's Note

All claims expressed in this article are solely those of the authors and do not necessarily represent those of their affiliated organizations, or those of the publisher, the editors and the reviewers. Any product that may be evaluated in this article, or claim that may be made by its manufacturer, is not guaranteed or endorsed by the publisher.
